# Ancestry Informative Marker Set for Han Chinese Population

**DOI:** 10.1534/g3.112.001941

**Published:** 2012-03-01

**Authors:** Hui-Qi Qu, Quan Li, Shuhua Xu, Joseph B. McCormick, Susan P. Fisher-Hoch, Momiao Xiong, Ji Qian, Li Jin

**Affiliations:** * School of Public Health, University of Texas Health Science Center at Houston, Brownsville, Texas 78520; † Endocrine Genetics Lab, McGill University Health Center (Montreal Children’s Hospital), Montréal, Québec H3H 1P3, Canada; ‡ Chinese Academy of Sciences and Max Planck Society (CAS-MPG) Partner Institute for Computational Biology, Shanghai Institutes for Biological Sciences, Chinese Academy of Sciences, Shanghai 200031, People’s Republic of China; § School of Public Health, University of Texas Health Science Center at Houston, Houston, Texas 77030; ** State Key Laboratory of Genetic Engineering and Ministry of Education (MOE) Key Laboratory of Contemporary Anthropology, School of Life Sciences and Institutes of Biomedical Sciences, Fudan University, Shanghai 200433, People’s Republic of China; †† China Medical City (CMC) Institute of Health Sciences, Taizhou, Jiangsu 225300, People’s Republic of China

**Keywords:** ancestry informative marker, Han Chinese, genetic association study, population structure

## Abstract

The population of Han Chinese is ∼1.226 billion people. Genetic heterogeneity between northern Han Chinese (N-Han) and southern Han Chinese (S-Han) has been demonstrated by recent genome-wide studies. As an initial step toward health disparities and personalized medicine in Chinese population, this study developed a set of ancestry informative markers (AIM) for Han Chinese population.

Han Chinese compose the largest ethnic group in the world, which accounts for 91.51% of the Chinese population, or ∼1.226 billion people, according to China’s 2010 census (http://www.chinadaily.com.cn/china/2011-04/28/content_12415449.htm). Chronic diseases including cancer, vascular disease, and infectious diseases, are the leading causes of death in this population (He *et al.* 2005). Genetic association study (GAS), a critical approach to understanding molecular mechanisms and population-specific genetic risk of these diseases, can lead to the development of effective interventions at an individual or population level. Currently, a major issue of GAS is the confounding effect of population stratification, which is a common source of false-positive or false-negative results in genetic association studies with case-control study design (Ziv and Burchard 2003). Our recent study identified obvious genetic heterogeneity between northern Han Chinese (N-Han) and southern Han Chinese (S-Han), historically divided by the natural barrier, the Yangtze River (Xu *et al.* 2009). This study highlighted the importance of the correction for population stratification in GAS of the Han Chinese population.

Population stratification is due to the presence of genetic subgroups with different allele frequencies within a population. When different population subgroups have different disease prevalence, the differences detected in allele frequencies between cases and controls might in fact be independent of disease etiology but actually related to different prevalence. They could result from the underlying sampling bias inherent in the unknown distribution of different genetic populations in the overall sample. This is a common reason for erroneous conclusions of disease associations (Cardon and Bell 2001). By correction for population stratification, a GAS will be able to eliminate spurious genetic associations and thus avoid further fruitless downstream efforts. In addition, a GAS may gain additional statistical power by correcting for population stratification, as shown by our previous study that showed that estimation of the genetic effect for candidate loci could be biased by population divergence (He *et al.* 2008). To correct for population stratification, structured association identifies subpopulations within the larger population and tests genetic associations conditioned by the inferred ancestral information (Pritchard and Donnelly 2001). This structured association approach represented by the Eigenstrat algorithm (Price *et al.* 2006) has been extensively demonstrated to be an effective approach for the correction of population stratification. To infer subpopulations, a number of DNA polymorphism markers are required with substantially different allele frequencies among the subpopulations, *i.e.* ancestry informative markers (AIM). The genotypes of a set of AIMs will enable the classification of subpopulations. To date, there is still no consensus standard to define the number of AIMs for correction of population stratification in each specific population. Genotyping cost is a major factor that determines the number of AIMs used in a study (Londin *et al.* 2010). We therefore developed a set of AIMs for genetic studies of Han Chinese populations. To minimize the genotyping cost of structured association studies, the classification performance of different number of AIMs were assessed.

## MATERIALS AND METHODS

This study analyzed a sample of 308 Han Chinese individuals from different geographic regions in China. In this sample, 150,916 autosome SNPs were genotyped with call rate >95% (Xu *et al.* 2009). Principal component analysis (PCA) implemented in the Eigenstrat software (Patterson *et al.* 2006; Price *et al.* 2006) was used to identify ethnic outliers and genetically admixed individuals. For the PCA analysis, 18,000 tag SNPs without obvious linkage disequilibrium (LD; r^2^ < 0.2) were selected genome-wide. Two-hundred thirty-six individuals were unambiguously distinguishable as N-Han or S-Han, and thus were selected for defining the AIMs in Han Chinese. Geographic distribution of these 236 individuals is described in Table 1. In these 236 individuals, ancestry information content *I_a_* of each autosome SNP was calculated using the *infocalc* program based on information-theoretic principles (Rosenberg *et al.* 2003). Across 22 autosomes, an initial set of AIMs including 5000 SNPs was selected by choosing one SNP marker with the largest *I_a_* in each 500 kb window. Each SNP marker has frequency >0.05 in both N-Han and S-Han, and has low LD (r^2^ < 0.2) with distance of >100 kb from the preceding AIM.

**Table 1:** Geographic distribution of the 236 Han Chinese individuals

Geographic Location	Number of Individuals	Historic Classification
Beijing	22	Northern Han
Gansu	13	Northern Han
Non-specific northern Han	9	Northern Han
Hebei	39	Northern Han
Heilongjiang	7	Northern Han
Henan	10	Northern Han
Jilin	3	Northern Han
Liaoning	4	Northern Han
Neimeng	7	Northern Han
Ningxia	2	Northern Han
Shandong	26	Northern Han
Shannxi	3	Northern Han
Shanxi	10	Northern Han
Tianjin	1	Northern Han
Xinjiang	6	Northern Han
Anhui	1	Southern Han
Guangdong	24	Southern Han
Guangxi	1	Southern Han
Hubei	2	Southern Han
Hunan	1	Southern Han
Jiangsu	11	Southern Han
Jiangxi	2	Southern Han
Shanghai	14	Southern Han
Sichuan	3	Southern Han
Yunnan	2	Southern Han
Zhejiang	13	Southern Han

## RESULTS AND DISCUSSION

To enable the application of AIMs in genetic studies of Han Chinese, these 5000 AIMs are listed in supporting information, Table S1, ranked by *I_a_*. Shown by the PCA using these 5000 AIMs, N-Han and S-Han individuals formed two obviously distinct clusters by the first principal component (PC1). This finding is concordant with a recent genome-wide SNP genotyping study that revealed a one-dimensional “north-south” population structure in Han Chinese population (Chen *et al.* 2009). For this initial set of AIMs of 5000 SNPs, *I_a_* of each SNP is highly correlated with its eigenvector weight of PC1 (r = 0.936; Figure S1). This evidence is further support that the information content *I_a_* is mainly determined by one-dimensional “north-south” population structure.

**Figure 1:**
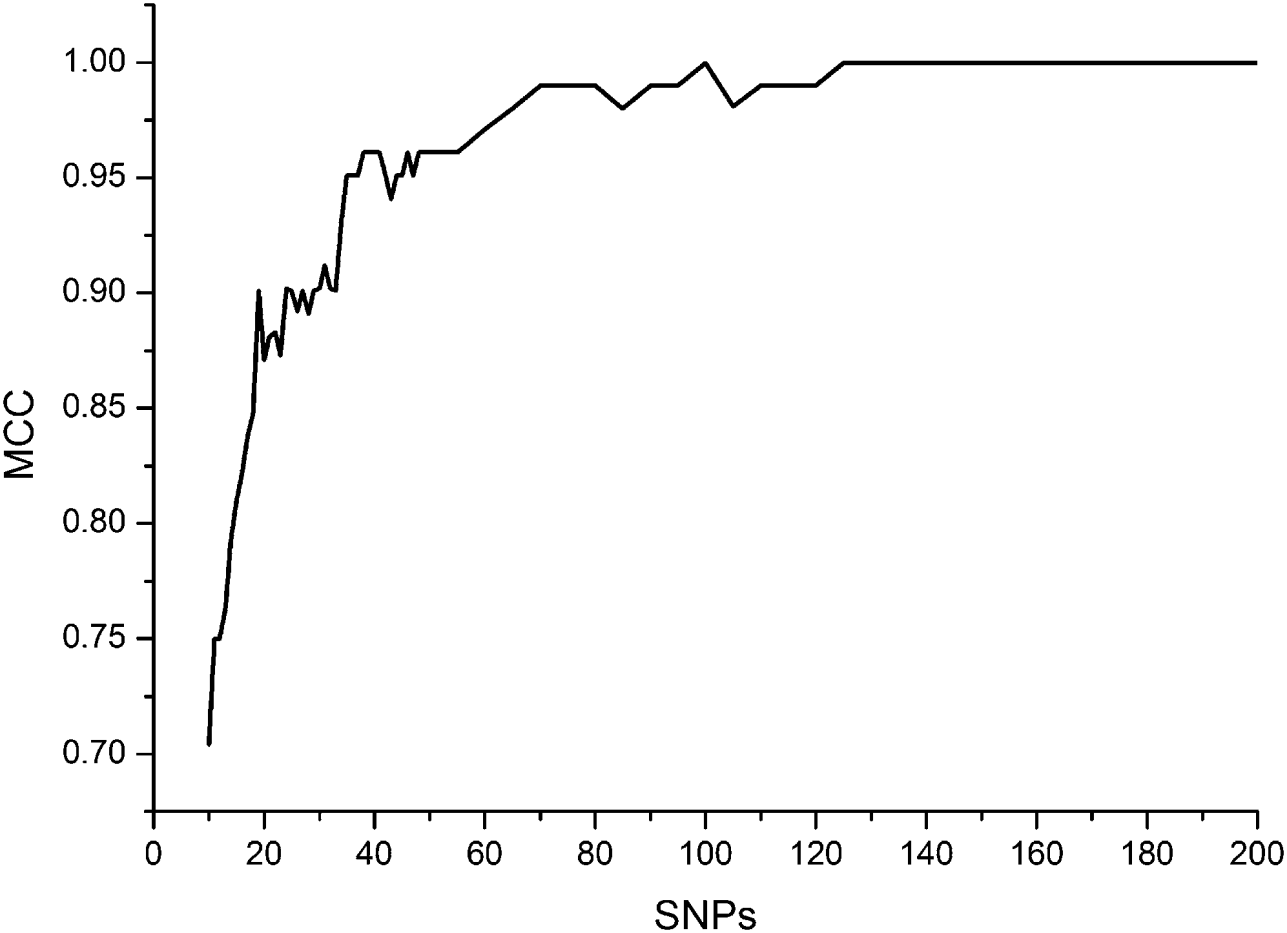
Maximum Matthews correlation coefficient (MCC) of principal component analysis (PCA) clustering using different number of ancestry informative markers (AIM). The clustering performance is compromised obviously when the number of AIMs decreases to 30. Horizontal axis: the number of SNPs with robust *I_a_*. Vertical axis: maximum MCC of each set of AIMs.

By a stepwise procedure, we decreased the number of AIMs and investigated the change of PCA clustering. In each step, we decreased the number of AIMs by removing AIMs with the smallest *I_a_*. The classification effect of PCAs was assessed by the maximum Matthews correlation coefficient (MCC) of each set of AIMs (Matthews 1975). We observed that the clustering effect was compromised significantly when less than 30 AIMs were used (Figure 1; Figure S2). On the basis of this analysis, we recommend at least the top 30 SNPs in the AIM list in Table S1 should be used in any structured association study on the Han-Chinese population. More robust correction for population stratification is expected when the top 140 AIMs in Table S1 are used, which differentiated N-Han and S-Han unambiguously in our study (Table 2). We further validated the performance of sets of AIMs by k-fold cross-validation. A threefold cross-validation achieved highly similar MCCs as the original model.

**Table 2:** Classification performance of different number of AIMs

Number of AIMs	PC1 Cutoff	MCC	Specificity	Sensitivity
15	−0.04	0.810	0.966	0.901
20	−0.03	0.871	0.930	0.952
25	−0.03	0.901	0.971	0.952
30	−0.02	0.902	0.932	0.969
35	−0.03	0.951	0.986	0.976
40	−0.03	0.961	0.986	0.982
45	−0.04	0.951	1.000	0.970
50	−0.03	0.961	1.000	0.976
60	−0.03	0.971	1.000	0.982
70	−0.03	0.990	1.000	0.994
80	−0.03	0.990	1.000	0.994
90	−0.02	0.990	0.987	1.000
100	−0.03	1.000	1.000	1.000
110	−0.04	0.990	1.000	0.994
120	−0.04	0.990	1.000	0.994
130	−0.03	1.000	1.000	1.000
140	−0.04	1.000	1.000	1.000
150	−0.03	1.000	1.000	1.000

AIM, ancestry informative marker; MCC, Matthews correlation coefficient; PC1, first principal component.

Difference in some common phenotypic traits, *e.g.* body height, facial features, and daily food compositions, are obvious between N-Han and S-Han Chinese. The population structure by genome-wide studies (Xu *et al.* 2009; Chen *et al.* 2009) highlighted the importance of correction for population stratification in genetic association study of Han Chinese. A large number of genetic studies are being performed in Han Chinese population, the majority being case-control studies. By providing a set of AIMs, our study aims to help to address the potential population stratification in genetic association studies. However, it is worth emphasizing that correction for population stratification may not always be addressed sufficiently using AIMs (Seldin and Price 2008). Replication of genetic association in an independent study is always important. Besides correction for population stratification, ancestry information inferred using the AIMs in Han Chinese may be used to assess genetic components underlying common traits, as differences in risk for some diseases have been observed between N-Han and S-Han Chinese (Rao *et al.* 2000; Zhao *et al.* 2004). Understanding subpopulation-specific risk factors for common diseases using the AIMs can be an initial step toward personalized medicine in the era of post-human genome projects (Barnes 2010).

## Supplementary Material

Supporting Information

## References

[bib1] Barnes K. C., 2010 Ancestry, ancestry-informative markers, asthma, and the quest for personalized medicine. J. Allergy Clin. Immunol. 126: 1139–1140 21134571 10.1016/j.jaci.2010.10.032

[bib2] Cardon L. R. Bell J. I., 2001 Association study designs for complex diseases. Nat. Rev. Genet. 2: 91–99 11253062 10.1038/35052543

[bib3] Chen J. Zheng H. Bei J.-X. Sun L. Jia W.-h., 2009 Genetic structure of the Han Chinese population revealed by genome-wide SNP variation. Am. J. Hum. Genet. 85: 775–785 19944401 10.1016/j.ajhg.2009.10.016 PMC2790583

[bib4] He J. Gu D. Wu X. Reynolds K. Duan X., 2005 Major causes of death among men and women in China. N. Engl. J. Med. 353: 1124–1134 16162883 10.1056/NEJMsa050467

[bib5] He Y. Jiang R. Fu W. Bergen A. W. Swan G. E., 2008 Correlation of population parameters leading to power differences in association studies with population stratification. Ann. Hum. Genet. 72: 801–811 18652602 10.1111/j.1469-1809.2008.00465.x PMC2574891

[bib6] Londin E. R. Keller M. A. Maista C. Smith G. Mamounas L. A., 2010 CoAIMs: a cost-effective panel of ancestry informative markers for determining continental origins. PLoS ONE 5: e13443 20976178 10.1371/journal.pone.0013443 PMC2955551

[bib7] Matthews B. W., 1975 Comparison of the predicted and observed secondary structure of T4 phage lysozyme. Biochim. Biophys. Acta 405: 442–451 1180967 10.1016/0005-2795(75)90109-9

[bib8] Patterson N. Price A. L. Reich D., 2006 Population structure and eigenanalysis. PLoS Genet. 2: e190 17194218 10.1371/journal.pgen.0020190 PMC1713260

[bib9] Price A. L. Patterson N. J. Plenge R. M. Weinblatt M. E. Shadick N. A., 2006 Principal components analysis corrects for stratification in genome-wide association studies. Nat. Genet. 38: 904–909 16862161 10.1038/ng1847

[bib10] Pritchard J. K. Donnelly P., 2001 Case-control studies of association in structured or admixed populations. Theor. Popul. Biol. 60: 227–237 11855957 10.1006/tpbi.2001.1543

[bib11] Rao X. Wu X. Folsom A. R. Liu X. Zhong H., 2000 Comparison of electrocardiographic findings between Northern and Southern Chinese population samples. Int. J. Epidemiol. 29: 77–84 10750607 10.1093/ije/29.1.77

[bib12] Rosenberg N. A. Li L. M. Ward R. Pritchard J. K., 2003 Informativeness of genetic markers for inference of ancestry. Am. J. Hum. Genet. 73: 1402–1422 14631557 10.1086/380416 PMC1180403

[bib13] Seldin M. F. Price A. L., 2008 Application of ancestry informative markers to association studies in European Americans. PLoS Genet. 4: e5 18208330 10.1371/journal.pgen.0040005 PMC2211545

[bib14] Xu S. Yin X. Li S. Jin W. Lou H., 2009 Genomic dissection of population substructure of Han Chinese and its implication in association studies. Am. J. Hum. Genet. 85: 762–774 19944404 10.1016/j.ajhg.2009.10.015 PMC2790582

[bib15] Zhao L. Stamler J. Yan L. L. Zhou B. Wu Y., 2004 Blood pressure differences between northern and southern Chinese: role of dietary factors: the International Study on Macronutrients and Blood Pressure. Hypertension 43: 1332–1337 15117915 10.1161/01.HYP.0000128243.06502.bc PMC6688605

[bib16] Ziv E. Burchard E. G., 2003 Human population structure and genetic association studies. Pharmacogenomics 4: 431–441 12831322 10.1517/phgs.4.4.431.22758

