# Supplementary material for: Ancestry Informative Marker Set for Han Chinese Population
Source: G3 (Bethesda). 2012 Mar 1;2(3):339–41. doi: 10.1534/g3.112.001941 (PMC3291503; doi:10.1534/g3.112.001941)
Supplement: Supporting Information [file supp_2_3_339__index.html]

Supporting Information 

# Ancestry Informative Marker Set for Han Chinese Population

## Supporting Information for Qu *et al*, 2012

**Files in this Data Supplement:**

- Supporting Information - Figures S1 and S2 and Table S1 (PDF, 868 KB)
- Figure S1 - Correlation of *Ia* and PC1 weight values of the 5,000 AIMs (PDF, 178 KB)
- Figure S2 - The clustering performance of different number of AIMs (PDF, 653 KB)
- Table S1 - The list of 5,000 AIMs ranked by *Ia* (.xlsx, 326 KB)
