# Supplementary material for: Ancestry Informative Marker Set for Han Chinese Population
Source: G3 (Bethesda). 2012 Mar 1;2(3):339–41. doi: 10.1534/g3.112.001941 (PMC3291503; doi:10.1534/g3.112.001941)
Supplement: Supporting Information [file supp_2.3.339_001941SI.pdf]

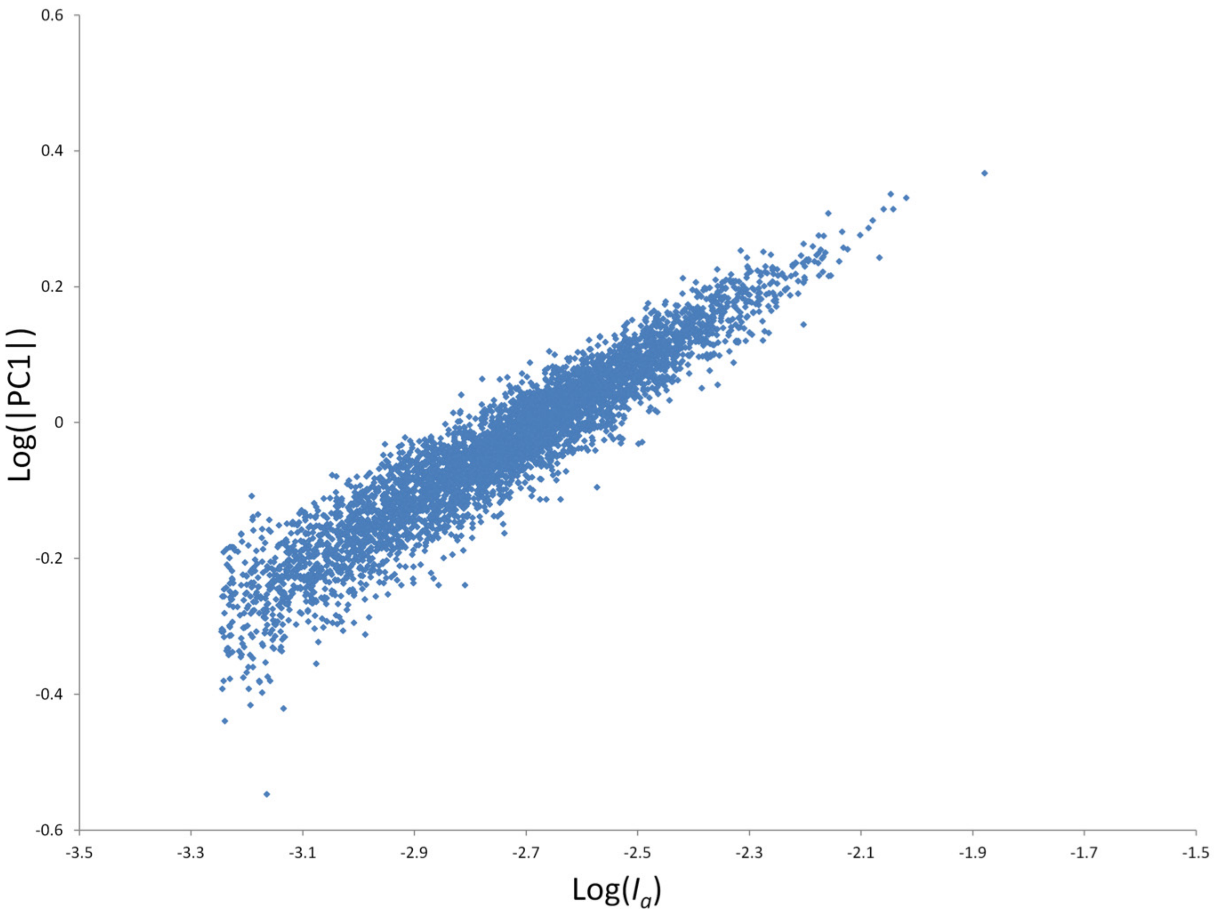

Figure S1 Correlation of I_a and PC1 weight values of the 5,000 AIMs. The PC1 weight values of the AIM markers were calculated using the “snpweightoutname“ function implemented in the EIGENSOFT package[16](http://genepath.med.harvard.edu/~reich/Software.htm). I_a of each of the 5,000 SNPs is highly correlated with its eigenvector weight of PC1 (r=0.947). The horizontal axis represents Log(I_a); The vertical axis represents Log(|PC1|).

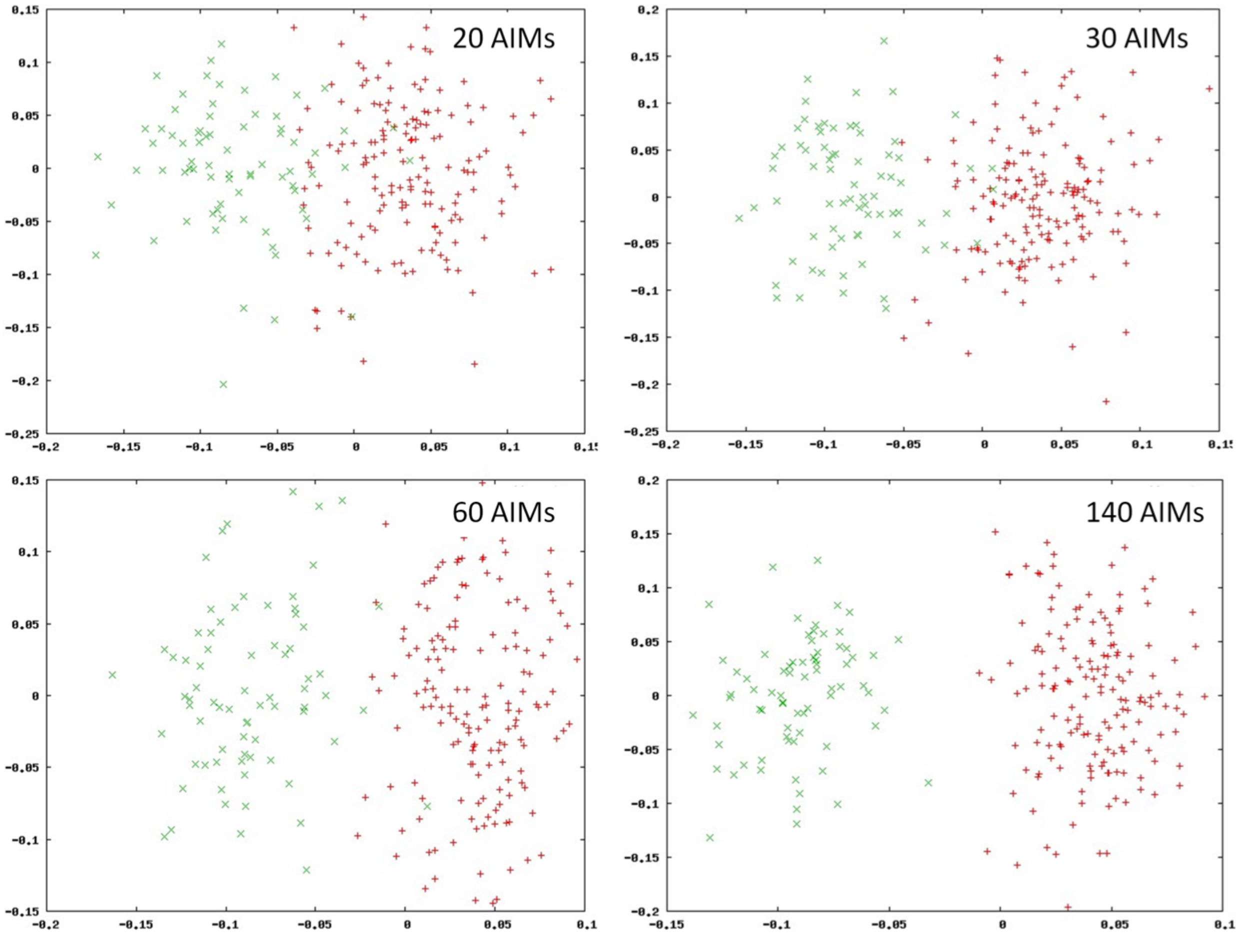

Figure S2 The clustering performance of different number of AIMs. Shown by this figure, S-Han and N-Han Chinese can be differentiated unambiguously when 140 AIMs are used. However, when the number of AIMs decreases to 30, the clustering performance of the AIMs is compromised obviously. Green markers represent S-Han Chinese; red markers represent N-Han Chinese. The horizontal axis represents PC1; The vertical axis represents PC2.

Table S1 The list of 5,000 AIMs ranked by I_a. To correct for population stratification, the priorities of AIMs decrease from the top to the bottom of this list.

Table S1 is available for download as an Excel file at http://www.g3journal.org/lookup/suppl/doi:10.1534/g3.112.001941/-/DC1.
