# Supplementary material for: Ancestry Informative Marker Set for Han Chinese Population
Source: G3 (Bethesda). 2012 Mar 1;2(3):339–41. doi: 10.1534/g3.112.001941 (PMC3291503; doi:10.1534/g3.112.001941)
Supplement: Supporting Information [file supp_2.3.339_FigureS1.pdf]

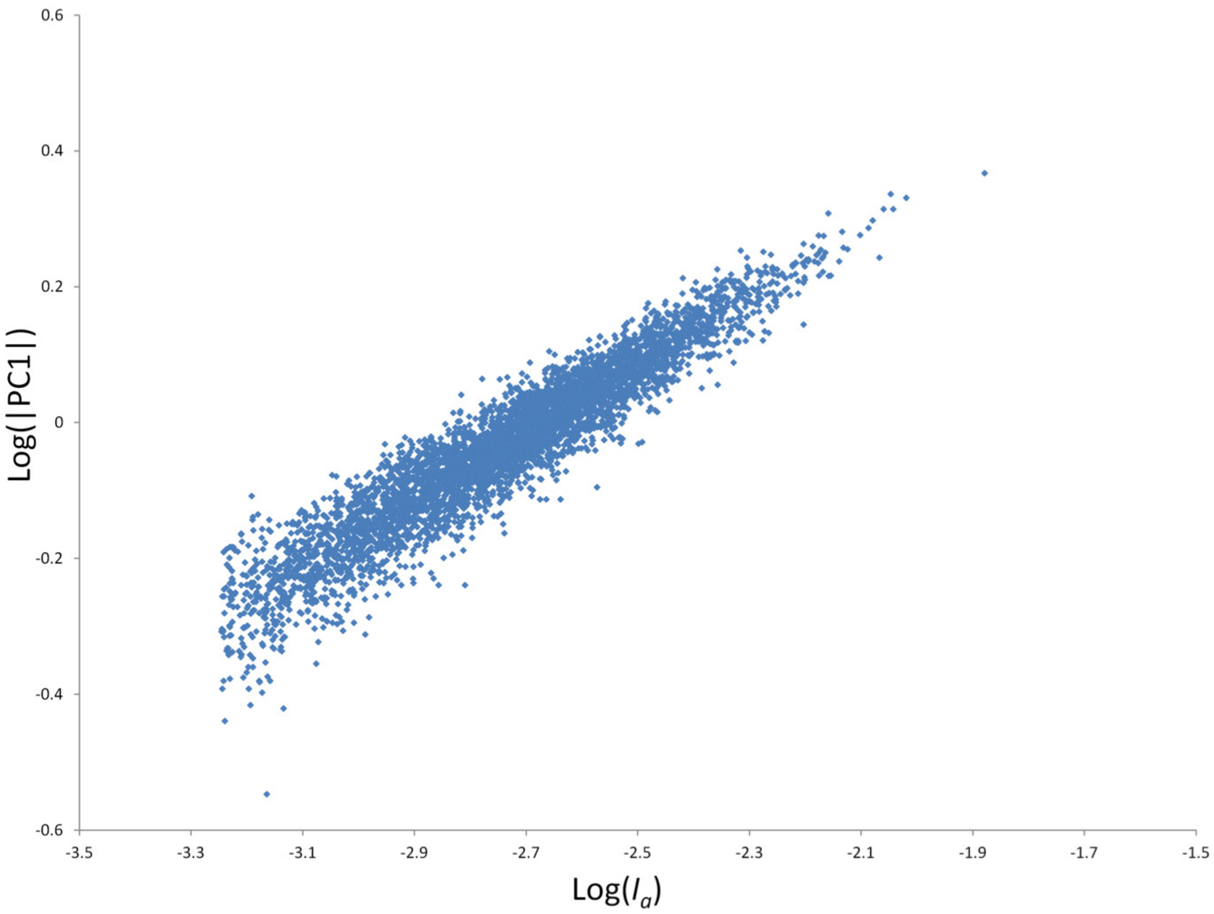

Figure S1 Correlation of I_a and PC1 weight values of the 5,000 AIMs. The PC1 weight values of the AIM markers were calculated using the "snpweightoutname" function implemented in the EIGENSOFT package[16](http://genepath.med.harvard.edu/~reich/Software.htm). I_a of each of the 5,000 SNPs is highly correlated with its eigenvector weight of PC1 (r=0.947). The horizontal axis represents Log(I_a); The vertical axis represents Log(|PC1|).
