# Supplementary material for: Ancestry Informative Marker Set for Han Chinese Population
Source: G3 (Bethesda). 2012 Mar 1;2(3):339–41. doi: 10.1534/g3.112.001941 (PMC3291503; doi:10.1534/g3.112.001941)
Supplement: Supporting Information [file supp_2.3.339_FigureS2.pdf]

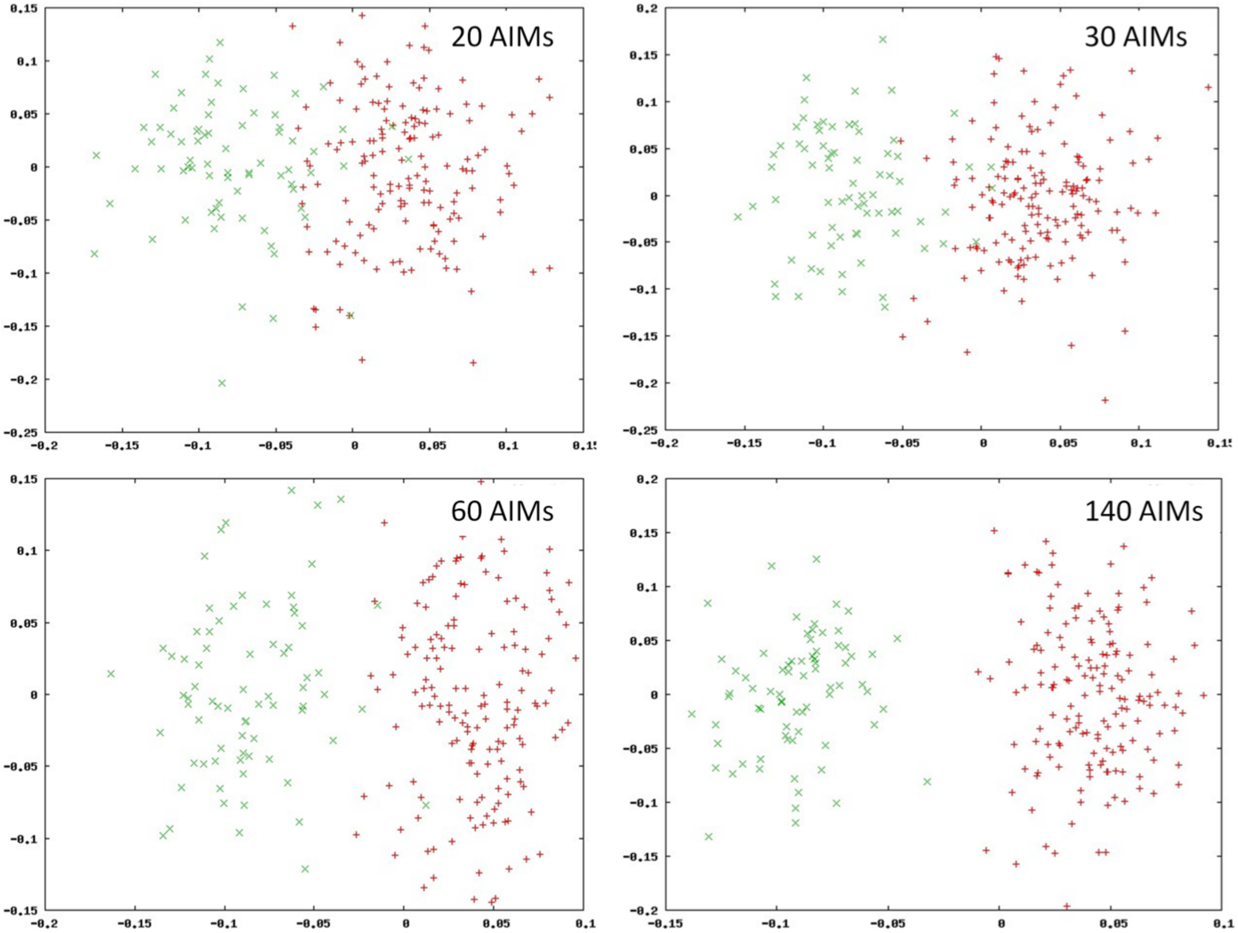

Figure S2 The clustering performance of different number of AIMs. Shown by this figure, S-Han and N-Han Chinese can be differentiated unambiguously when 140 AIMs are used. However, when the number of AIMs decreases to 30, the clustering performance of the AIMs is compromised obviously. Green markers represent S-Han Chinese; red markers represent N-Han Chinese. The horizontal axis represents PC1; The vertical axis represents PC2.
